# Feasibility study of 4D-online monitoring of density gradients induced by lung cancer treatment using carbon ions

**DOI:** 10.3389/fonc.2025.1502960

**Published:** 2025-02-26

**Authors:** Claire-Anne Reidel, Enrico Pierobon, Felix Horst, Lévana Gesson, Athena Paz, Christian Graeff, Timo Steinsberger, Klemens Zink, Matthias Witt, Yannick Senger, Christian Finck, Marie Vanstalle, Chiara La Tessa, Marco Durante, Uli Weber, Christoph Schuy

**Affiliations:** ^1^ Biophysics Department, GSI Helmholtzzentrum für Schwerionenforschung GmbH, Darmstadt, Germany; ^2^ UNITN-TIFPA, University of Trento, Trento Institute for Fundamental Physics and Applications, Trento, Italy; ^3^ Université de Strasbourg, CNRS, IPHC UMR 7871, Strasbourg, France; ^4^ Institute of Electrical Engineering and Information Technology, Technische Universität Darmstadt, Darmstadt, Germany; ^5^ Institute of Medical Physics and Radiation Protection (IMPS), University of Applied Sciences, Giessen, Germany; ^6^ Marburg Ion-Beam Therapy Center MIT, Marburg, Germany; ^7^ Radiation Oncology Department, University of Miami, Miami, FL, United States; ^8^ Institute of Condensed Matter Physics, Technische Universität Darmstadt, Darmstadt, Germany; ^9^ Department of Physics “Ettore Pancini”, University Federico II, Naples, Italy

**Keywords:** 4D monitoring, charged particle detection, CMOS pixel detector, moving target, vertices reconstruction, high density gradient

## Abstract

Tumor motion is a major challenge for scanned ion-beam therapy. In the case of lung tumors, strong under- and overdosage can be induced due to the high density gradients between the tumor- and bone tissues compared to lung tissues. This work proposes a non-invasive concept for 4D monitoring of high density gradients in carbon ion beam therapy, by detecting charged fragments. The method implements CMOS particle trackers that are used to reconstruct the fragment vertices, which define the emission points of nuclear interactions between the primary carbon ions and the patient tissues. A 3D treatment plan was optimized to deliver 2 Gy to a static spherical target volume. The goodness of the method was assessed by comparing reconstructed vertices measured in two static cases to the ones in a non-compensated moving case with an amplitude of 20 mm. The measurements, performed at the Marburg Ion-Beam Therapy Center (MIT), showed promising results to assess the conformity of the delivered dose. In particular to measure overshoots induced by high density gradients due to motion with 83.0 ± 1.5% and 92.0 ± 1.5% reliability based on the ground truth provided by the time-resolved motor position and depending on the considered volume and the iso-energy layers.

## 1 Introduction

Advanced beam delivery techniques, such as raster scanning, combined with, e.g., carbon ion beams, are nowadays used for cancer therapy to deliver a highly conformal dose distribution (1, 2). However, geometric changes between the treatment planning CT images and the patient anatomy during the treatment can induce under- and overdosage in the tumor and the healthy tissues, respectively. Geometrical variations such as patient movement, positioning uncertainties and organ motion are considered by applying additional margins to the clinical target volume and by using, e.g., robust optimization. Inter- and intrafractional motions are a major cause for dose degradation, therefore, mitigation strategies were extensively studied (3–5). Intrafractional motion, the focus of the present study, is induced by the respiration of the patient, and represents a great challenge in scanned particle therapy. For lung cancer, intrafractional motion can induce severe range uncertainties due to strong density gradients between lung and tumor/bone tissues. In the study carried out by (6), the tumor motion was monitored via time-resolved computer tomography (4DCT), in the range 0–23 mm with an average amplitude of 10 mm. According to (5), motion mitigation techniques can be classified in two categories: prevent or reduce anatomical changes, and adapt the treatment planning or delivery. To reduce the impact of motion, breath-hold techniques (7–9) and abdominal press (10) were investigated, however, these methods can be uncomfortable, especially for patients with advanced lung cancer who are physically unable to use the breath-hold technique. External motion tracking employing cameras, monitoring the surface movement of the patient (11), and internal tumor tracking by means of fiducial markers detected by electromagnetic/optical sensors or fluoroscopy (12, 13), were investigated for respiratory correlated imaging. Mitigation and tracking of the motion are typically combined with gating, which delivers the beam at a specific phase of the respiratory cycle (14), and rescanning, which delivers the beam several times over the same spot to improve the dose homogeneity (15). Several studies for 4D-robust optimization, which is incorporated in the treatment plan, were performed to consider the intrafractional motion of the tumor (16, 17). Although different strategies are used to improve the dose conformity for intrafractional motion, real-time monitoring techniques are of great interest to verify the delivered dose and potentially directly mitigate motion. Recent studies were performed for magnetic resonance imaging (MRI)-guided radiotherapy (18) and for proton range monitoring using the thermoacoustic pressure waves emitted by thermal expansion of the dose deposition (19). The use of a mixed helium and carbon ion beam was also proposed, to simultaneously perform imaging via the highly penetrating helium ions and treatment via the carbon ions (20). Several alternative methods for real-time monitoring use secondary particles emitted by the interaction of the primary beam with the patient. The detection of prompt gammas, created by nuclear interactions between the primary ion beam and the tissues, was investigated for proton beam range monitoring, employing different detection techniques, and is currently assessed in clinical routine (21–23). Positron emission tomography (PET) was investigated to monitor the range of proton or carbon ion beams by detecting the 511 keV photon pairs, created by the annihilation of the produced positron emitters (24, 25). Nevertheless, the limited amount of statistics and the biological washout degrade the spatial resolution of the reconstructed images. An innovative study for PET applications was conducted to resolve these issues by using radioactive beams, e.g., ^11^C or ^15^O, directly for the treatment (26). However, their complex production and comparatively low intensities are a major hindrance toward clinical application and different solutions are currently investigated in several projects (27).

Another technique for range monitoring, which is most relevant for the scope of this work, is the interaction vertex imaging (IVI) technique, originally proposed by (28). This method aims to reconstruct the trajectories and emission points of the secondary charged particles produced during nuclear fragmentation between the primary ions and the patient tissues. These light fragments, mainly protons and helium ions, have a longer range than the primary carbon ions and are emitted at larger angles with respect to the primary beam (29). Previous studies investigated the relation between the interaction point distribution and the position of the Bragg peak for online range monitoring (30–33). However, these studies were performed with single pencil beams and for a number of primary ions higher than delivered during a typical patient treatment. Another study, using the IVI technique for lateral pencil beam position monitoring, performed the measurements for an anthropomorphic phantom with a clinical-like treatment fraction (34). Additionally, a recent study was performed to detect interfractional changes in the patient anatomy using IVI (35). Furthermore, first realistic, clinical prototype systems were developed, implemented and tested based on the IVI technique (36, 37).

The purpose of the present work is to propose and assess a concept for 4D-online monitoring of high density gradients applied to a simplified lung tumor case, where the tumor tissue density (∼ 1 g/cm^3^) is more than four times higher than the surrounding lung tissue (∼ 0.23 g/cm^3^). In this study, the IVI technique is used to reconstruct the vertex distributions in *x*-*y*-*z* of the secondary charged particles produced during nuclear fragmentation of primary carbon ion beams interacting with the phantom volume. The number of produced lighter fragments in a defined volume varies as a function of its density. This feature is exploited to detect density gradients induced by a phantom composed of different materials representing the tumor/bone and lung tissues for several static cases, and finally, for cases simulating intrafractional motion. In the case that the treatment is delivered as planned, the vertex distributions of the different carbon ion beams have certain profiles in *x*-*y*-*z*. However, in the case of motion during the treatment, the strong density gradients induced by, e.g., tumor tissues compared to lung tissues can induce notable range shifts and, therefore, the vertex distributions show differences.

In this work, the phantom simulating tumor and lung tissues, was irradiated with a clinical-like spherical treatment plan of 2 Gy and clinical-like particle intensities. The respiratory motion was simulated by a remote controlled motorized table. Four complementary metal–oxide–semiconductor (CMOS) pixel sensor particle trackers were placed behind the phantom at several angles with respect to the beam axis, and the vertices were reconstructed for different scenarios. First, a statistical analysis was performed to evaluate the number of reconstructed vertices compared to the number of planned primary carbon ions. Second, the vertex distributions were computed for all recorded measurements and presented for the different scenarios. Finally, the high density gradients induced by the spherical edge of the target were studied and the method efficiency was assessed as a function of the scanned beam spots. In addition, the feasibility of detecting the motion phase of the target was studied using the proposed concept.

## 2 Materials and methods

### 2.1 Experimental setup

**Figure 1:**
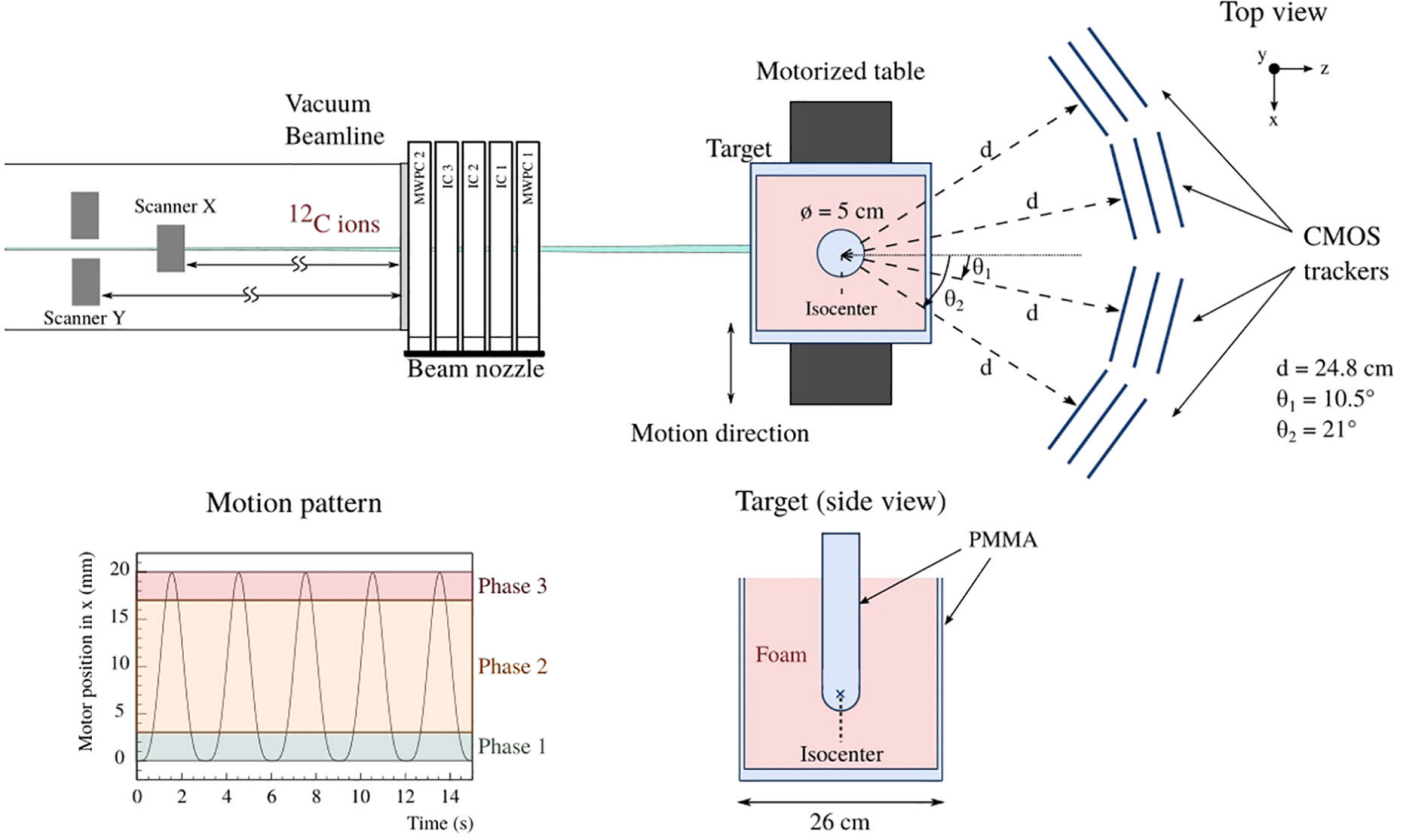
A schematic of the experimental setup comprising twelve MIMOSA-28 pixel sensors used to monitor high density gradients from a simulated moving tumor is shown (top). Additionally, the motor position subdivided in the three used motion phases (bottom left), as well as a side view of the target (bottom right) are represented.

The measurements were performed with carbon ion beams at the Ion-Beam Therapy Center in Marburg (MIT), Germany (38). The experimental setup is depicted in **Figure 1**, and the different parts of the setup are detailed in the following sections.

#### 2.1.1 Moving target phantom

The target was composed of a polymethylmethacrylate (PMMA) container, referred to as aquarium, of 26×26×26 cm^3^, filled with a 24×25×25 cm^3^ foam material of 0.23 g/cm^3^ density to represent the lung tissue. A PMMA cylinder of 5 cm diameter and 1.17 g/cm^3^ density was manufactured with a spherical end. The PMMA volume was inserted at the center of the foam material, representing the tumor and lung tissues, respectively. The phantom was positioned on a motorized table, controlled by a software designed to reproduce the respiratory motion of the patient (39). The table was initially positioned to match the center of the PMMA spherical end to the isocenter of the treatment room, defined as the zero coordinates (referred to as static_in) in this work. Two static cases were measured first, where the phantom was on one hand centered (static_in), and on the other hand at the extreme position of the motion of 20 mm (referred to as static_out). The moving case was then measured following a motion pattern as proposed by Lujan et al. (40) of 3 s period and 20 mm amplitude (**Figure 1**). For the present study, the motion cycle was split in three phases with respect to the amplitude, where Phase 1, Phase 2 and Phase 3 cover the range 0–3 mm, 3–17 mm and 17–20 mm, respectively. Movement of the motorized table was started by a trigger signal provided by the treatment control system when the beam delivery started. Upon activation, the internal system of the motor recorded the time and position with around 100 µs resolution.

#### 2.1.2 MIMOSA-28 sensor

The MIMOSA-28 (Minimum Ionizing MOS Active pixel sensor) is a pixel sensor fabricated in Austria Micro Systems (AMS)-0.35 µm CMOS process with an active area of around 2×2 cm^2^ and composed of 928 rows × 960 columns of squared pixels with a length of 20.7 µm. The sensor has a total thickness of 50 µm including an epitaxial layer of 14 µm. It employs line by line readout and delivers a digital signal within an integration time of 186.5 µs (∼ 5 kHz frame rate) (41).

When a particle passes through the sensor, the created charges are collected by one or several pixels and a binary output is obtained from pixels with a signal above a certain threshold set by the user. The analysis software QAPIVI (31), based on the ROOT (42) and GEANT4 (43) libraries, reconstructs groups of fired pixels, also called clusters, where the center of mass defines the cluster position. A straight line, called track, is reconstructed by matching the clusters of the different sensors of a tracker, comprising typically three CMOS pixel sensors. The individual vertices, defined as the position of the interaction point from, e.g., nuclear fragmentation, are then computed using the minimum calculated distance between the average extrapolated track per beam spot in front of the target, provided by the multi-wire proportional chambers (MWPCs) of the nozzle, and the reconstructed back projected track from the CMOS tracker behind the target. In order to reach high track resolution, it is necessary to align the trackers using a dedicated low intensity run without target to compensate mechanical mispositioning of the sensors via a software alignment procedure (44). A detailed explanation of the vertex reconstruction procedure is given in Section 2.2.1.

#### 2.1.3 CMOS tracker systems

The experimental setup for this study comprised a total of twelve MIMOSA-28 pixel sensors, where two sets of six sensors each were read out by two independent data acquisition (DAQ) systems. Four trackers composed of three sensors each were positioned at different angles behind the phantom. A preliminary study, using Monte Carlo simulations with GEANT4 (43), was performed to estimate the resolution uncertainty of the reconstructed vertices as a function of the tracker distance and angle compared to the target center, as in (31). The simulations were performed for 170 MeV/u carbon ions interacting with the center of the target in *x* and *y* and the primary energy was chosen to fully stop the primary ions at the center of the PMMA cylinder along the beam axis. In this work, the optimized distances and angles of the trackers were chosen as a compromise between the mechanical constraints and the Monte Carlo simulation predictions. The CMOS trackers were positioned behind the target at ±10.5° and ±21° with respect to the beam axis, and the radial distance from tracker- to target center was set to 24.8 cm.

#### 2.1.4 Treatment planning

**Figure 2:**
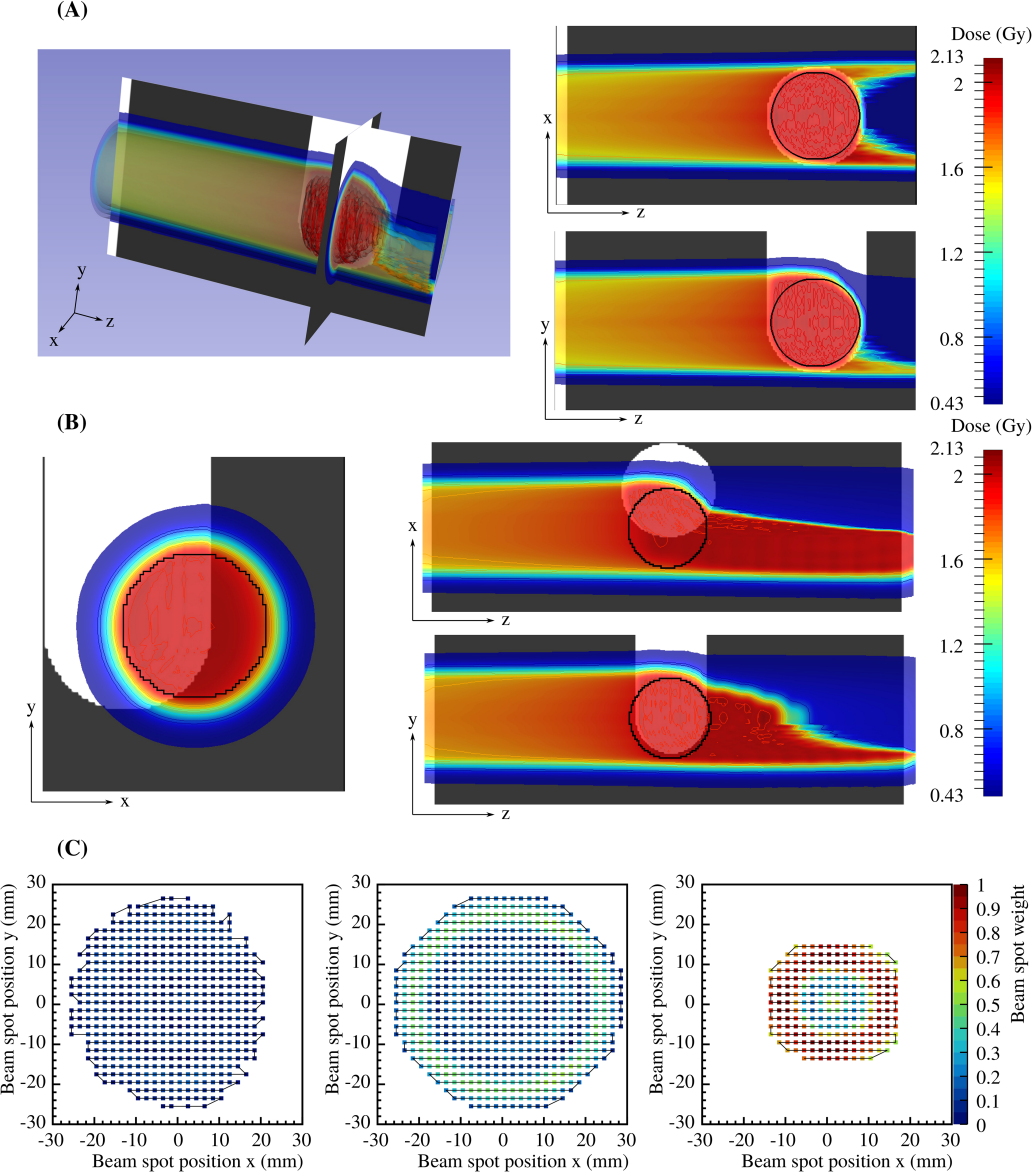
Delivered dose superimposed to the synthetic CT as 2D and 3D views when the treatment is delivered as planned **(A)**, while in **(B)** the treatment is delivered at a motor position of 20 mm in *x* direction. In **(C)**, *x*-*y* views of three single iso-energy layers that were extracted from the 3D treatment plan for 131.32, 170.11 and 195.65 MeV/u containing 495, 603 and 219 beam spots, respectively. The black line shows the trajectory of the irradiation, and the particles per beam spot were normalized to the maximum value of the 195.65 MeV/u treatment plan.

Treatment plans for all investigated scenarios were computed with the treatment planning system TRiP (Treatment planning for particles), which was originally developed during the pilot project for carbon ion therapy at the GSI Helmholtzzentrum für Schwerionenforschung, Germany (45). The treatment plans were calculated based on a synthetic CT, where the geometry and the density of the different components were set to reproduce the phantom used during the experiment. The volume of interest was defined as a sphere fitting the spherical end of the PMMA cylinder, with margins 3 mm smaller than the spherical PMMA volume. The inner margins were included to compensate, e.g., misalignment during the experiment. The treatment plan of the sphere was computed for the base data of MIT for carbon ions, and optimized for a homogeneous physical dose of 2 Gy in the volume of interest. In **Figure 2A**, the planned dose superimposed to the synthetic CT is shown as 2D and 3D views. The treatment planning resulted in 18 iso-energy layers ranging from 125.91 to 203.69 MeV/u with a total of 8360 beam spots. In **Figure 2B**, the three 2D views of the delivered dose at a motor position of 20 mm are shown, and strong under- and overdosage can be observed. In **Figure 2C**, an *x*-*y* view of three single iso-energy layers, extracted from the 3D treatment plan, is presented for 131.32, 170.11 and 195.65 MeV/u containing 495, 603 and 219 beam spots, respectively. Each point in panel (C) represents a single beam spot and was normalized to the maximum value of the 195.65 MeV/u iso-energy layer. Three simplified sub-plans were calculated for these single iso-energy layers and scaled-up to around 2 Gy to better understand and disentangle the data from the more complex 3D treatment plan.

#### 2.1.5 Primary beam monitoring

The nozzle detectors of the treatment room, composed of two MWPCs and three ionization chambers (ICs), were used to control and monitor the treatment delivery, beam position and intensity, respectively, as well as to provide the average track information per beam spot in front of the phantom. Two pairs of dipole magnets were used to deflect the beam in *x* and *y*, referred to as Scanner X and Scanner Y in **Figure 1** at a distance of 7.534 m and 8.234 m, respectively, downstream of the isocenter position. The logfiles of each delivery were recorded for later use in the data reconstruction. They contain the scanner positions, the center of mass and the beam width in *x* and *y* measured by the two MWPCs, the number of particles for each spot, as well as the beam energy and a timestamp for each beam position.

#### 2.1.6 Setup synchronization

A Terasic DE0-Nano-SoC development board equipped with an Altera Cyclone V SE system-on-chip fieldprogrammable gate array (FPGA) was used to synchronize the different components of the experimental setup, via four signals provided by either the treatment control system or the individual data acquisition systems. The DAQ for the MIMOSA-28 can only synchronize six sensors, therefore, the readout frame clocks of two independent systems were sent to the FPGA. Additionally, the signal triggering the start of the motorized table as well as the next beam spot signal, where the number in the FPGA was incremented every time the beam spot moved, were recorded. All signals were timestamped by the internal FPGA clock of 100 kHz. Position deviations of the motorized table were monitored via the motor logfiles for consecutive runs and deviations were typically smaller than 25 µm.

The three different experimental cases (static_in, static_out and moving case) were irradiated with the four described treatment plans. The measurements were repeated at least five times in order to verify the feasibility and reliability of the measurement concept proposed in the present work.

### 2.2 Analysis procedure

#### 2.2.1 Vertex reconstruction

In a first step, the CMOS pixel sensor data were analyzed independently of the other systems. Each tracker was locally and globally aligned using the dedicated alignment run and all recorded tracks were reconstructed for the four individual trackers. Next, all files were merged based on the timing signals logged by the FPGA. Each reconstructed track from the CMOS sensors was back projected to the target, referred to as secondary track, and associated to the extrapolated front track based on the center of mass of the primary carbon ion beam spot measured by the two nozzle MWPCs, and is referred to as primary track. Due to the readout time differences between the nozzle detectors and the CMOS sensors, multiple individually measured secondary tracks by the CMOS trackers are assigned to a single primary track per beam spot measured by the MWPCs. Finally, the minimum distance between the primary and secondary tracks was determined. The coordinates *x*-*y*-*z* of the averaged position of the two tracks where the distance is minimum was then defined as the reconstructed vertex. For this, two reconstruction methods were studied: a) The vertices were computed after extrapolation of the primary and secondary tracks to the target volume, and b) The intersection point was computed based on a more realistic primary beam distribution and several corrections, taking into account multiple Coulomb scattering calculated via the Highland approximation (46, 47).

For both methods, the primary track was initially obtained by extrapolating a line of coordinates (*x_i_*, *y_i_*, *z_i_*) where *x_i_* and *y_i_* are the initial values of the primary beam position defined as the mean value of the center of mass measured by MWPC 1 and MWPC 2, and *z_i_* the position of MWPC 1 along the beam axis. The initial slope $a_{x_{i}}$ and $a_{y_{i}}$ were computed from the positions of the scanner magnets $z_{x_{m}}$ and $z_{y_{m}}$ where the position in *x_m_* and *y_m_* are zero, extrapolated to the voxel position in the target defined by the treatment plan (*x_v_*, *y_v_*, *z_v_*). For method b), the position of the primary particle track ( $x_{i}^{\prime }$ , $y_{i}^{\prime }$ ) was first randomized following a Gaussian function of mean values ( $x_{i}$ , $y_{i}$ ) and sigma values ( $\sigma_{x_{i}}$ , $\sigma_{y_{i}}$ ) measured by the nozzle MWPCs. The initial slope $a_{x_{i}}^{\prime }$ and $a_{y_{i}}^{\prime }$ were computed after adding the randomized multiple Coulomb scattering of the different layer materials, such as the air, the foam and the PMMA materials, calculated using the Highland approximation. The secondary particle tracks were extrapolated to the target position and blurred by taking into account the multiple scattering of the foam and PMMA layers of the phantom.

#### 2.2.2 Vertex distributions

A statistical analysis was performed by determining the number of reconstructed vertices compared to the number of delivered carbon ions, extracted from the treatment plan for the three experimental cases (static_in, static_out and moving case) of the three single iso-energy layer plans, as a function of the position in *x* and *y*. The number of primary ions and reconstructed vertices are presented as the average value of the five repeated measurements and the uncertainties are defined as their root mean square (RMS). It is important to note, that a minimum of 50 reconstructed vertices per spot was considered to give reasonable results and was used as a threshold in the following analysis.

To evaluate the differences of the studied cases, and detect high density gradients of the moving target, the vertex distributions in *x*-*y*-*z* were computed for each scanned beam spot and the computed vertices in *x*-*y*-*z* were extracted from the vertex distributions. The vertex distribution in *z* was established for positions within the aquarium length, excluding the vertices in front and behind the aquarium. The computed vertices in *x* and *y* were defined as the mean value of the Gaussian-like distributions, whereas the computed vertices in *z* were defined for a certain integral value of the vertex distribution. Integral values ranging from 50 to 100% of the total integral of the vertex distribution were computed, and the corresponding position in *z* of the integral value was then extracted. The final computed vertices in *z* were selected for the integral value where the difference between the static_in and static_out case was maximum. This was done for both reconstruction methods, and the difference between the static_in and static_out case was calculated for all beam spots. In this work, the results showing all reconstructed vertices will be referred to as vertex distributions *v_dist_*, while the computed vertex in *z* as a function of the number of the scanned beam spot is determined according to the integral of *v_dist_*, and will be referred to as computed vertex *v_computed_*.

#### 2.2.3 Comparison of the static and moving cases

The difference between the static_in and static_out case were calculated for the three single iso-energy layer plans and for the 3D treatment plan. For each treatment plan, all *v_computed_* were computed as the average value of the five repeated measurements for all scanned beam spots. The uncertainties were defined as the RMS extracted from the different measurements, and the total uncertainties of the difference between the static_in and static_out case were computed after quadratically summing the uncertainties of each case. To compare the goodness of both reconstruction methods, the global mean value and RMS of *v_computed_* over all scanned beam spots were calculated for the static_in case, as well as for the differences between the static_in and static_out case for the single iso-energy layer plans. In addition, *v_computed_* for the static_in and static_out case were compared to the planned Bragg peak positions in CT coordinates of the 3D treatment plan computed for both cases.

To analyze the moving case, *v_computed_* for the static_in and static_out cases were considered as references. The differences of the computed vertices in *z* between the moving case compared to the static cases were computed for all beam spots and will be referred to as difference_in and difference_out, respectively. A threshold value, referred to as *thr_diff_* was calculated based on the RMS of the computed vertex differences between the two static cases for the repeated measurements, and used to evaluate the significance of the differences.

#### 2.2.4 Detection of edge density gradients

**Figure 3:**
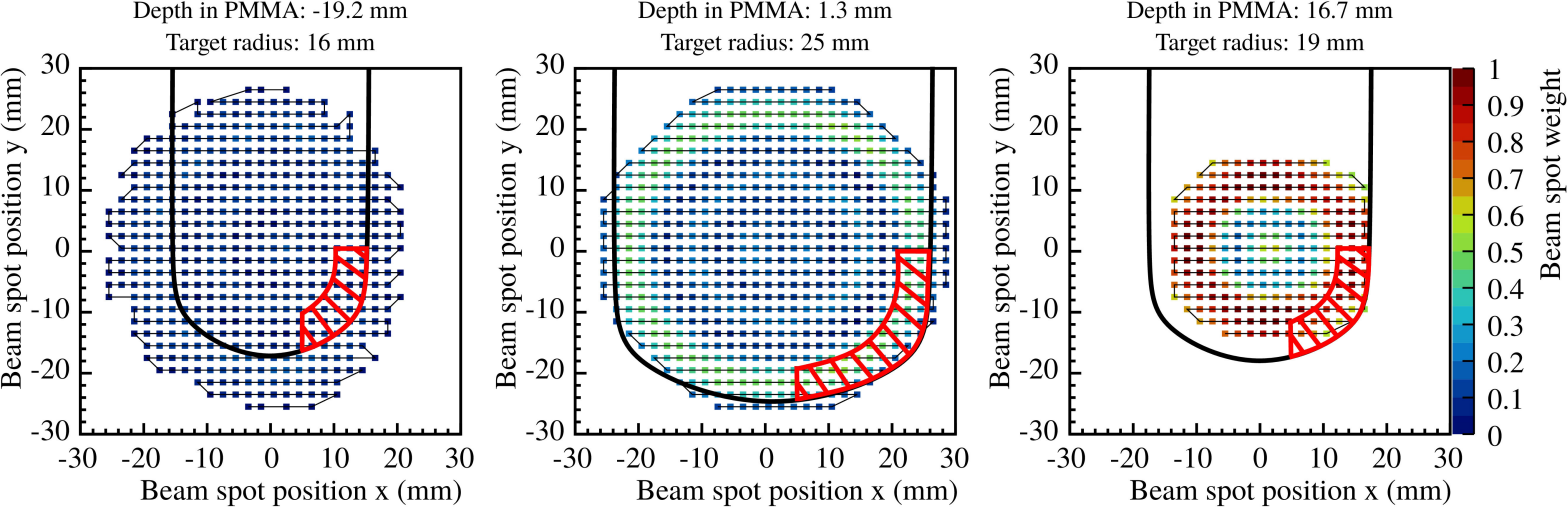
Selected area for single iso-energy layers for the study of edge density gradients due to motion, represented by the red dashed area. Panels (left to right) show the selected area for 131.32, 170.11 and 195.65 MeV/u, respectively.

To study the impact of sharp density gradients induced by material edges due to motion, the beam spots delivered to a defined target volume were selected and analyzed. The selected volume was chosen as a slice of 6 mm thickness following the shape of the spherical end of the target. Only the beam spots of positions bigger than 5 mm in *x* and smaller than 0 mm in *y* were selected in order to consider beam spots that traverse high density gradients due to motion. In **Figure 3**, the selected area for the three single iso-energy layers is depicted in red, containing 23, 45 and 26 beam spots for 131.32, 170.11 and 195.65 MeV/u, respectively. For the 3D treatment plan, the goodness of the detection method for edge density gradients induced by motion was assessed for all iso-energy layers, containing 610 points. The difference_in of the selected beam spots were compared to *thr_diff_*, mentioned in the previous section, and set to 2·RMS. The beam spots, where difference_in was below *thr_diff_*, were considered as delivered as planned, while the ones above the threshold were considered as not delivered as planned. In order to verify if the treatment was delivered as planned or not, the difference_in were subdivided to specific intervals based on the motor position, and referred to as *pos_m_*, varying between 0.5 and 3.5 mm. The goodness of the method was defined by the total efficiency, which corresponds to the ratio of the correctly categorized beam spots (delivered as planned or not) compared to the true number of beam spots in this category, assessed by the motor logfile.

#### 2.2.5 Detection of motion phase

Four different cases were defined and used to categorize each computed computed vertex *v_computed_* to one of the three motion phases represented in **Figure 1**. In case difference_in was below the threshold *thr_diff_* and difference_out above, vertices were assigned to Phase 1, corresponding to a high similarity to the static_in case. Given the opposite, vertices were assigned to Phase 3, corresponding to a high similarity to the static_out case. In case difference_in and difference_out were above *thr_diff_*, vertices were assigned to Phase 2, corresponding to an in–between state. Finally, all vertices with difference_in and difference_out below *thr_diff_* were not assigned to any motion phase and will be referred to as undefined.

To evaluate the reliability of the present analysis, the predicted cases from the computed vertices, associating *v_computed_* to a certain motion phase, were compared to the true motion phase provided by the motor logfile.

## 3 Results

### 3.1 Fragment tracking efficiency

**Figure 4:**
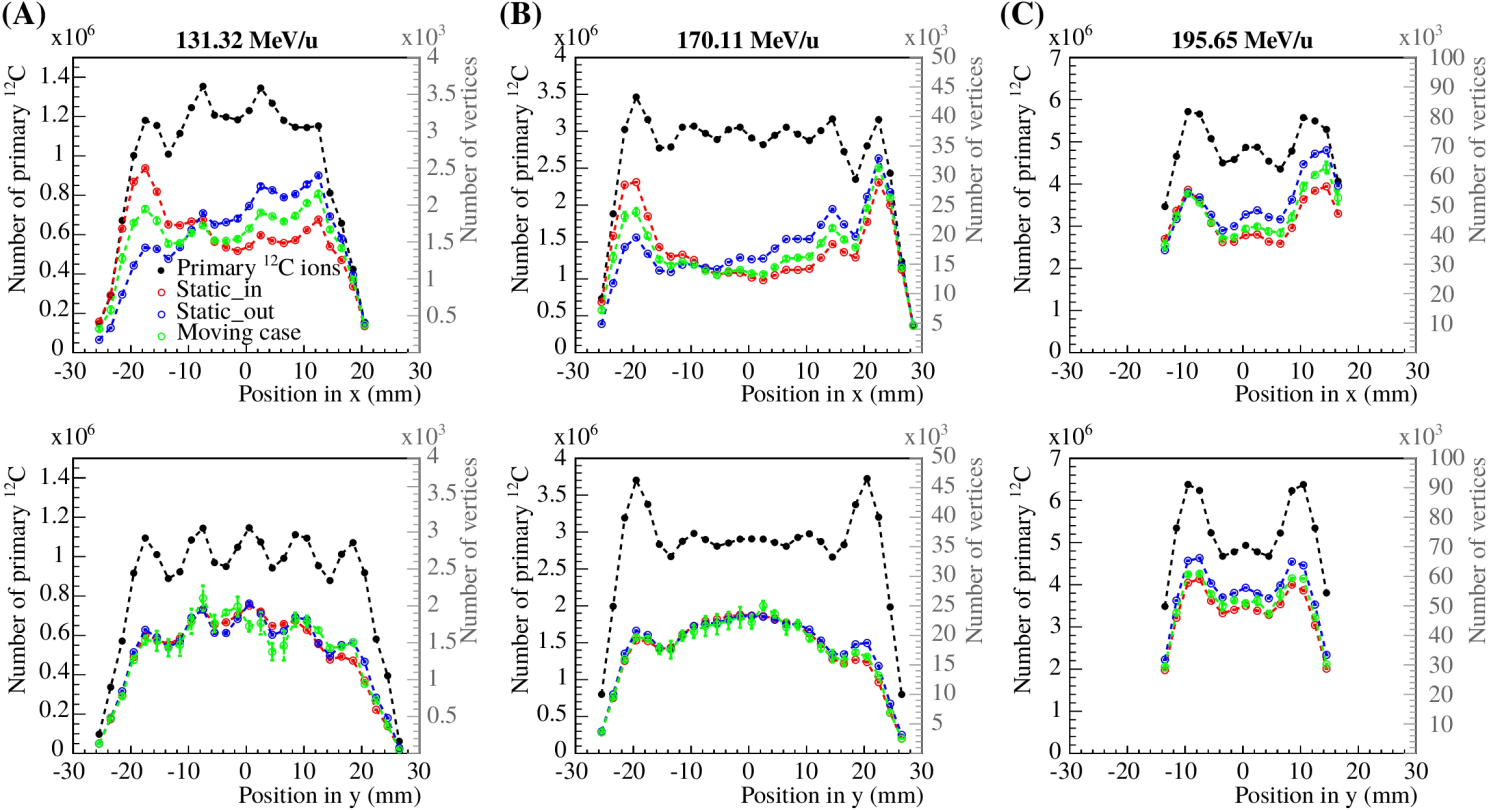
Statistical analysis of the reconstructed vertices compared to the number of delivered carbon ions from the treatment plan as a function of the beam spot position in *x* and *y*, integrated over *y* and *x*, respectively. Panels **(A–C)** show the results for 131.32, 170.11 and 195.65 MeV/u carbon ions, respectively. The left axis corresponds to the primary ions (black circles), while the right axis corresponds to the reconstructed vertices for the static_in, static_out and moving cases (blue, red and green empty circles).

The number of reconstructed vertices compared to the number of delivered primary carbon ions as a function of the position in *x* and *y* as given by the treatment plan, integrated over *y* and *x*, respectively, are shown in **Figure 4** for the three single iso-energy layer treatment plans.

The percentage of reconstructed vertices compared to the number of primary ions, for the experimental setup shown in **Figure 1**, was around 0.2, 0.7 and 1% in *x* and *y* for 131.32, 170.11 and 195.65 MeV/u carbon ions, respectively. For lower energy ion beams, the amount of detected fragments is smaller compared to higher energy beams, which is mainly due to the higher probability for light fragments to be stopped in the phantom before reaching the detectors. In addition, the four tracker angles were optimized for a pencil beam stopping at the center of the target, therefore, the tracking efficiency is higher for certain carbon ion beams. The data shows that the number of reconstructed vertices exhibit stronger differences along the *x*-axis, the motion direction of the target. Since the trackers were placed around the *y*-axis only, the uncertainties are larger for the *y*-position. The number of reconstructed vertices in *x* is larger for the static_out and moving cases, where the primary particles do not hit the PMMA cylinder. It is important to note that the total number of reconstructed vertices was computed from five independent repeated measurements. However, for the moving case, the position of the motor as a function of the primary beam delivery varied, resulting in a fluctuation of the number of reconstructed vertices, represented by the larger error bars.

### 3.2 Vertex distributions

**Figure 5:**
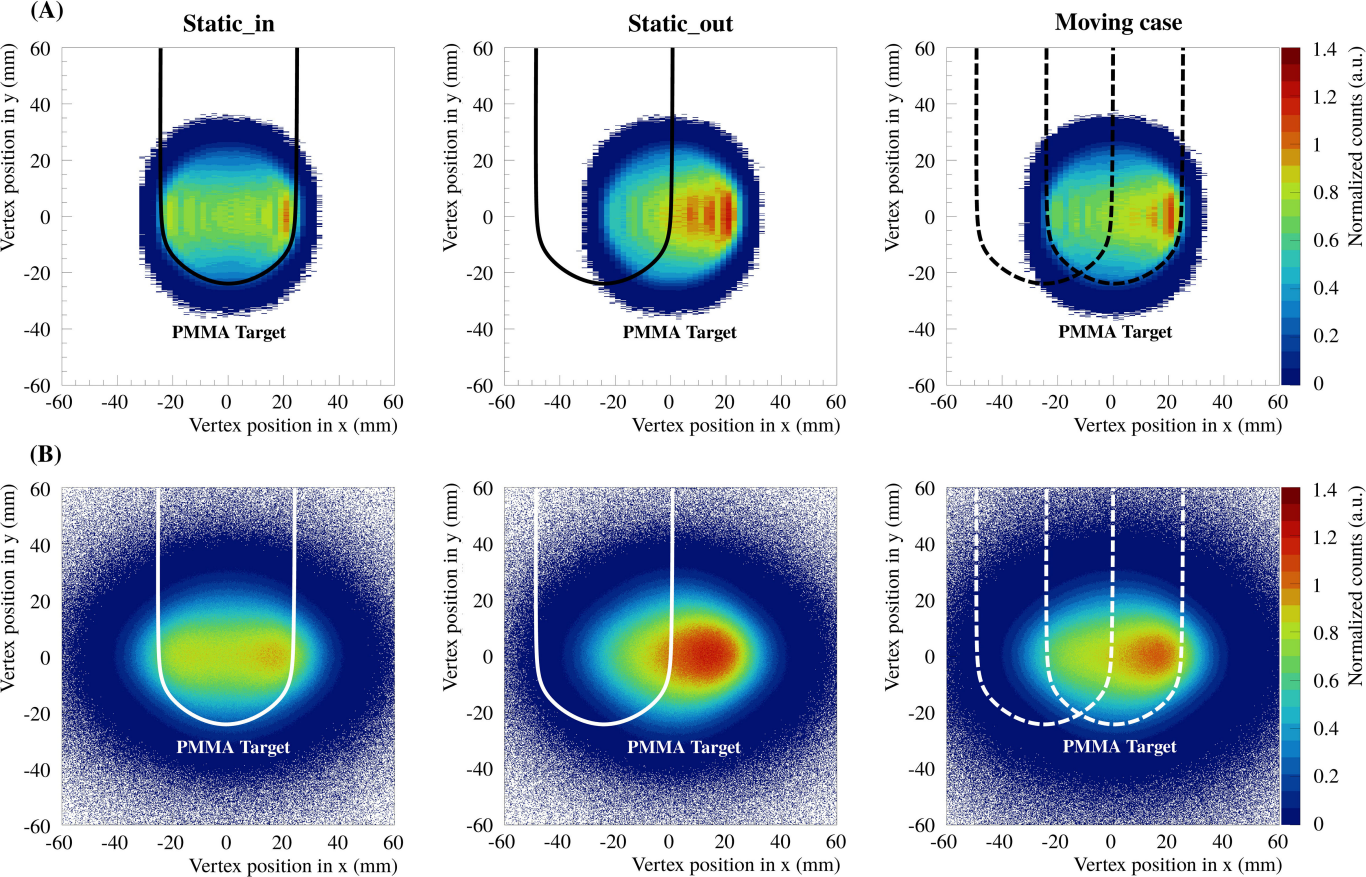
The 2D integrated profiles of all vertex distributions in *x*-*y* integrated over *z*, for the delivered 3D treatment plan and the three experimental cases (static_in, static_out and moving cases). Panels **(A, B)** show the results for the two reconstruction methods a) and b), respectively. The solid lines indicate the fixed position of the PMMA target, whereas the dashed lines indicate the extreme positions of the moving case.

**Figure 6:**
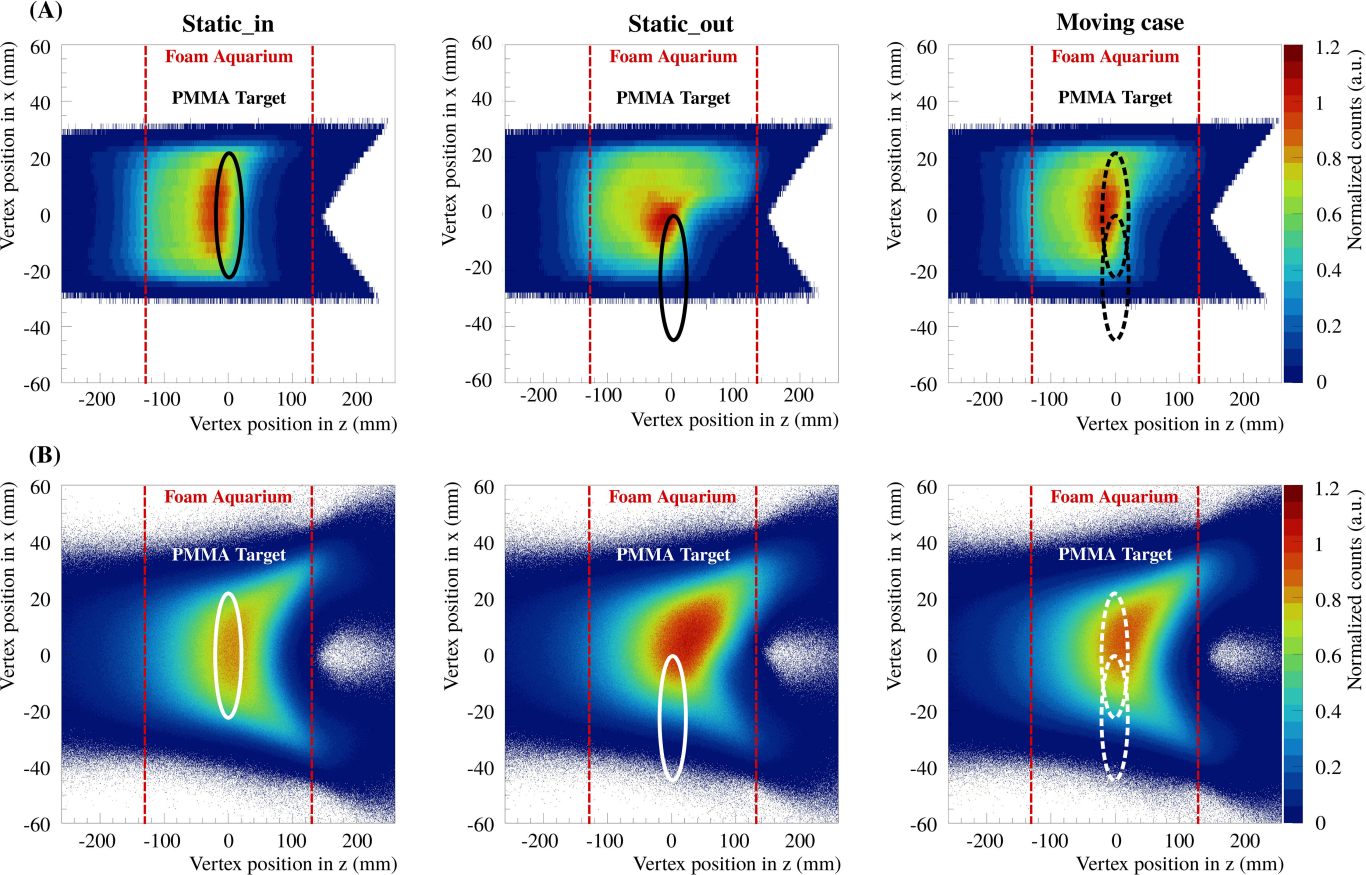
The 2D integrated profiles of all vertex distributions in *x*-*z* integrated over *y*, for the delivered 3D treatment plan and the three experimental cases (static_in, static_out and moving cases). Panels **(A, B)** show the results for the two reconstruction methods a) and b), respectively.

All vertices in *x*-*y*-*z* of each detected particle were reconstructed for the different measurements described prior for both reconstruction methods with and without multiple scattering, referred to as methods a) and b) in Section 2.2.1, respectively. In **Figures 5**, **6**, the vertex distributions of the 3D treatment plan are shown in the planes *x*-*y* and *x*-*z* after integrating in *z* and *y*, respectively. For visibility, the bin intervals of the vertex positions in *x*-direction for reconstruction method a) was increased. The resulting vertex distributions in the plane *y*-*z* is not shown but is very similar to **Figure 5**. In each case, the vertices are shown for both reconstruction methods and for the three experimental cases (static_in, static_out and moving cases). The 2D integrated profiles were normalized to the maximum value of the static_in case.

**Figure 7:**
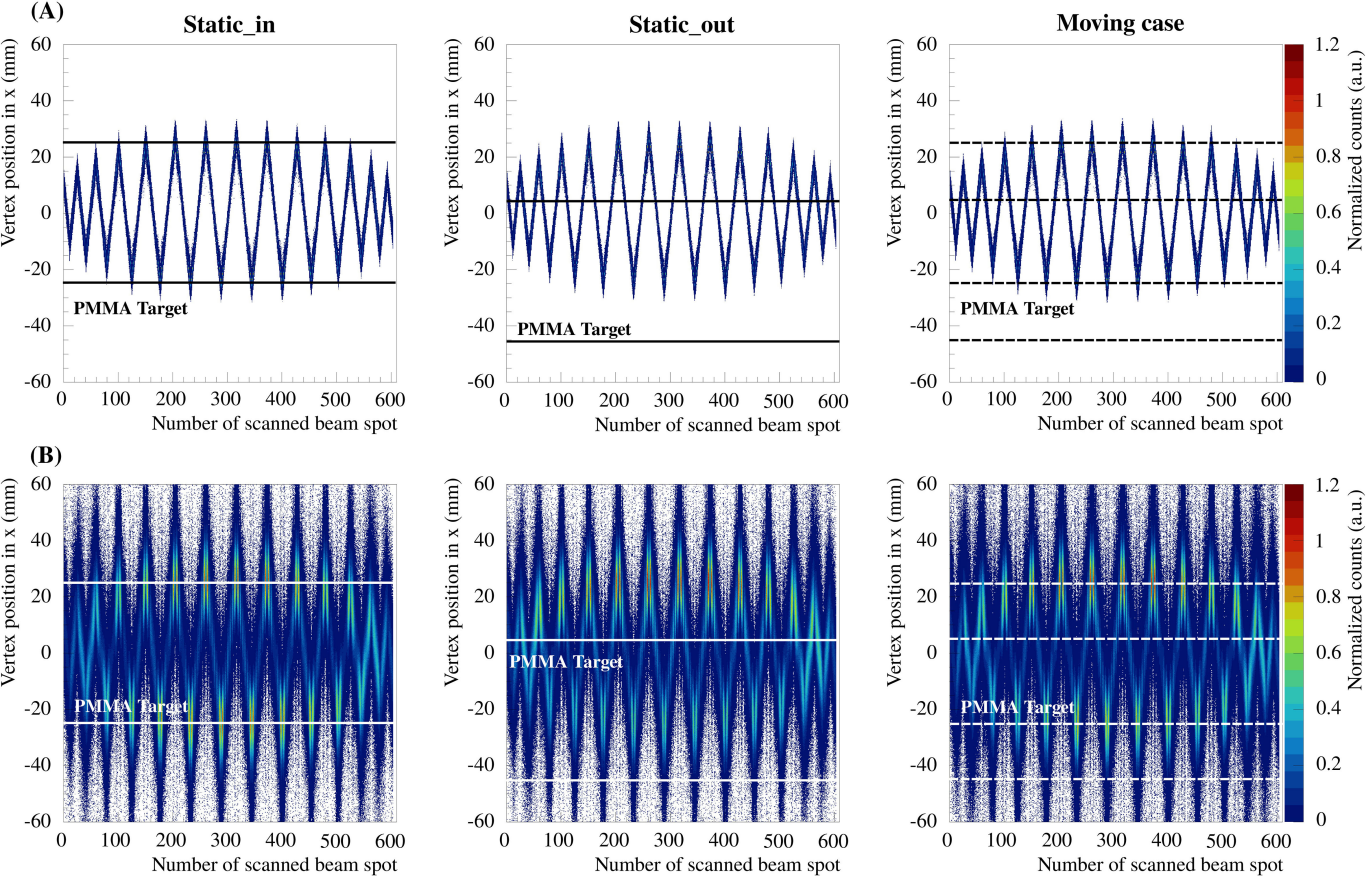
Vertex distributions in *x* integrated over *y* and *z* as a function of the scanned beam spot number, for the iso-energy layer treatment plan of 170.11 MeV/u carbon ions and the three experimental cases (static_in, static_out and moving cases). Panels **(A, B)** show the results for the two reconstruction methods without and with taking into account the multiple Coulomb scattering, respectively.

The 2D integrated profiles show that the high density gradients of the phantom yield significant differences between the static_in, static_out and the moving case. For the static_out and moving cases, the overshoot, in terms of number of reconstructed vertices, in the region where the PMMA cylinder was missed is around 30% and 20% compared to static_in, respectively. In addition, the range of the produced fragments increases. It can be seen that the overshoot from the treatment plan is also visible in the experimental results. For the present study, it is important to compute the vertices as a function of the treatment delivery timing in order to evaluate the feasibility of measuring high density gradients fast enough to detect tumor motion. In **Figure 7**, the vertex distributions in *x* as a function of the number of scanned beam spots are shown for the iso-energy layer treatment plan of 170.11 MeV/u carbon ions. The vertices are shown for both reconstruction methods and for the three experimental cases. The 2D integrated profiles were normalized to the maximum value of the static_in case.

The results presented in **Figure 7** show that the vertex distributions in *x* and *y* are similar for the different cases in terms of position, due to the fact that the reconstructed vertex positions in *x* and *y* are strongly influenced by the position of the primary ion beam. In all cases, the reconstructed results from method b) taking into account the primary beam distribution as well as the multiple Coulomb scattering show a more realistic case than the ones without. The results of the more simple reconstruction, following method a), are much sharper since the *x* and *y* positions of the primary reconstructed track were defined as the center of mass of the Gaussian beam spot.

### 3.3 Computed vertex comparison

**Figure 8:**
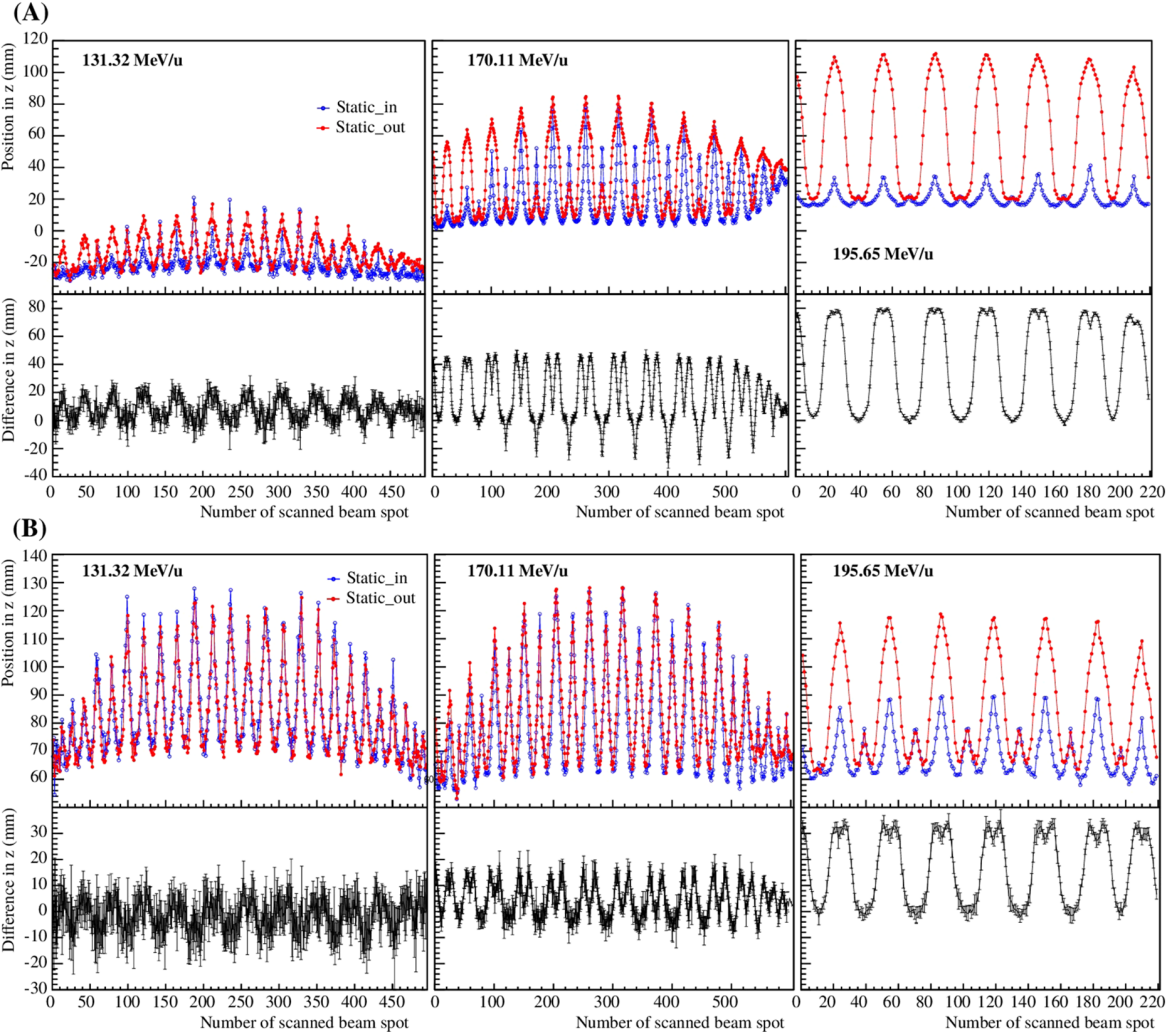
Computed vertex in *z* for the static_in and static_out cases and their difference as a function of the number of scanned beam spots for the three single iso-energy layer treatment plans (131.32, 170.11 and 195.65 MeV/u). Panels **(A, B)** correspond to the reconstruction methods a) and b), respectively.

The differences of the computed vertices in *z* were studied by comparing the static_in and static_out cases. As described in Section 2.2.2, the computed vertex *v_computed_* was extracted from the vertex distribution *v_dist_* for each single beam spot for a certain integral value. Since this work intends to detect strong differences from the vertex positions, the integral value where the difference in *z* is maximum was selected, resulting in 90% and 85% of the integral of the vertex distribution for the reconstruction methods a) and b), respectively. In **Figure 8**, the computed vertices of the static_in and static_out cases, as well as their difference, are shown as a function of the number of scanned beam spots for the three iso-energy layer treatment plans (131.32, 170.11 and 195.65 MeV/u). The zero position refers to the center of the target. The results were computed following the description in Section 2.2.3. For better visibility, the error bars in **Figure 8** are only shown for the computed vertex differences.

The results are summarized in **Table 1**, where $\bar{v_{computed}}$ is the mean value for the static_in case and $\bar{v_{diff}}$ the one of the differences between the static_in and static_out cases, and were calculated as the average value of all scanned beam spots. The RMS values are shown in parenthesis, and the results are presented for the three iso-energy layer plans and both reconstruction methods. The mean value $\bar{v_{computed}}$ is not linear as a function of the primary beam energy because of the filtering of the produced fragments due to their likely energies and due to the setup geometry. In addition, the setup was optimized for detecting fragments from primary carbon ions that stop at the center of the target. It can be seen that $\bar{v_{computed}}$ and $\bar{v_{diff}}$ are significantly bigger and better distinguished for different energy layers for the reconstruction method without taking into account multiple Coulomb scattering, which blurs the results. Since the present study focuses on strong differences, the results presented in the following sections will only be computed for the reconstruction method a).

**Table 1:** Mean value $\bar{v_{computed}}$ for the static_in case, as well as differences $\bar{v_{diff}}$ between the static_in and static_out cases.

Reconstruction method a)		
Energy (MeV/u)	$\bar{v_{computed}}$ (mm)	$\bar{v_{diff}}$ (mm)
131.32	-20.7 (8.6)	7.6 (7.2)
170.11	19.0 (16.7)	16.9 (19.3)
195.65	20.4 (5.1)	40.7 (31.7)

Reconstruction method b)		
Energy (MeV/u)	$\bar{v_{computed}}$ (mm)	$\bar{v_{diff}}$ (mm)
131.32	83.8 (15.7)	-1.4 (5.3)
170.11	78.7 (18.1)	4.4 (6.2)
195.65	68.8 (7.8)	16.8 (13.3)

The RMS values are shown in parenthesis. The results are shown for the three single iso-energy layer treatment plans, and for the two reconstruction methods a) and b).

**Figure 9:**
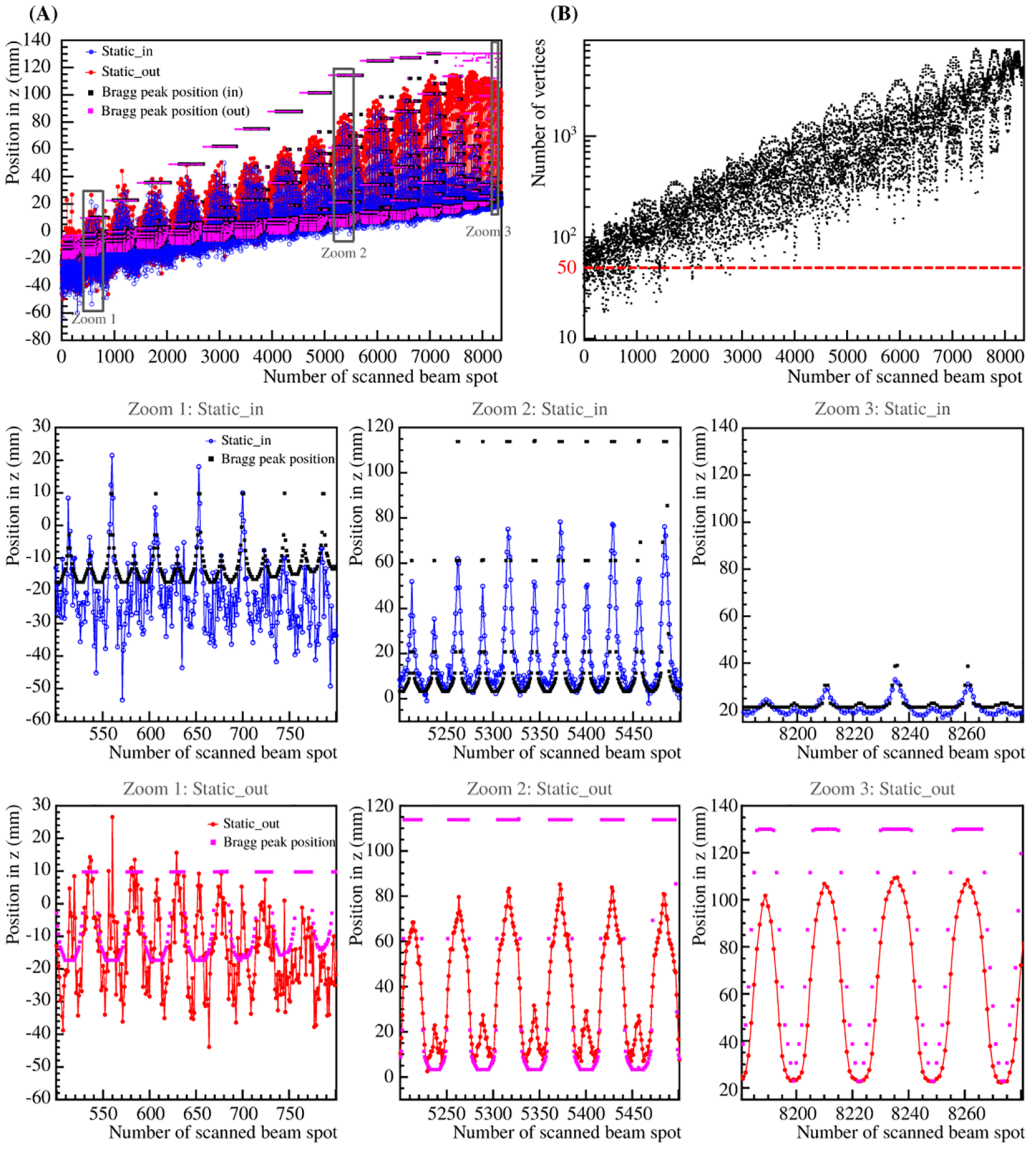
Computed vertex in *z* for the static_in and static_out cases superimposed on the planned Bragg peak position in CT coordinates of static_in and static_out cases **(A)**, as well as the number of reconstructed vertices **(B)** as a function of the number of scanned beam spots for the 3D treatment plan. For visibility, three zooms are shown for the static_in and static_out cases independently.

In **Figure 9**, the computed vertex *v_computed_* and the planned Bragg peak position in CT coordinates as a function of the number of scanned beam spots are shown for the 3D treatment plan for the static_in and static_out. The number of reconstructed vertices as a function of the number of scanned beam spots are also shown. As for the iso-energy layer plans, the results are shown after determining the computed vertices averaged from the five repeated measurements. For better visibility, three zoom windows were computed to highlight the regions where the energies are 131.32, 170.11 and 195.65 MeV/u corresponding to Zoom 1, Zoom 2 and Zoom 3, respectively.

The differences between the static_in and static_out cases depend on the primary beam energy, which influences the range overshoot in the foam volume. From **Figure 9B**, it is clear that the number of reconstructed vertices per scanned beam spot is small and precise range monitoring is not possible. However, since the method focuses on detecting high density gradients, a low amount of reconstructed vertices can be good enough to assess the target motion. In addition, it is possible to correlate the planned Bragg peak position in CT coordinates to the computed vertex in *z*, which can be used as additional information to monitor the treatment delivery.

### 3.4 Detection of edge density gradients for moving targets

**Figure 10:**
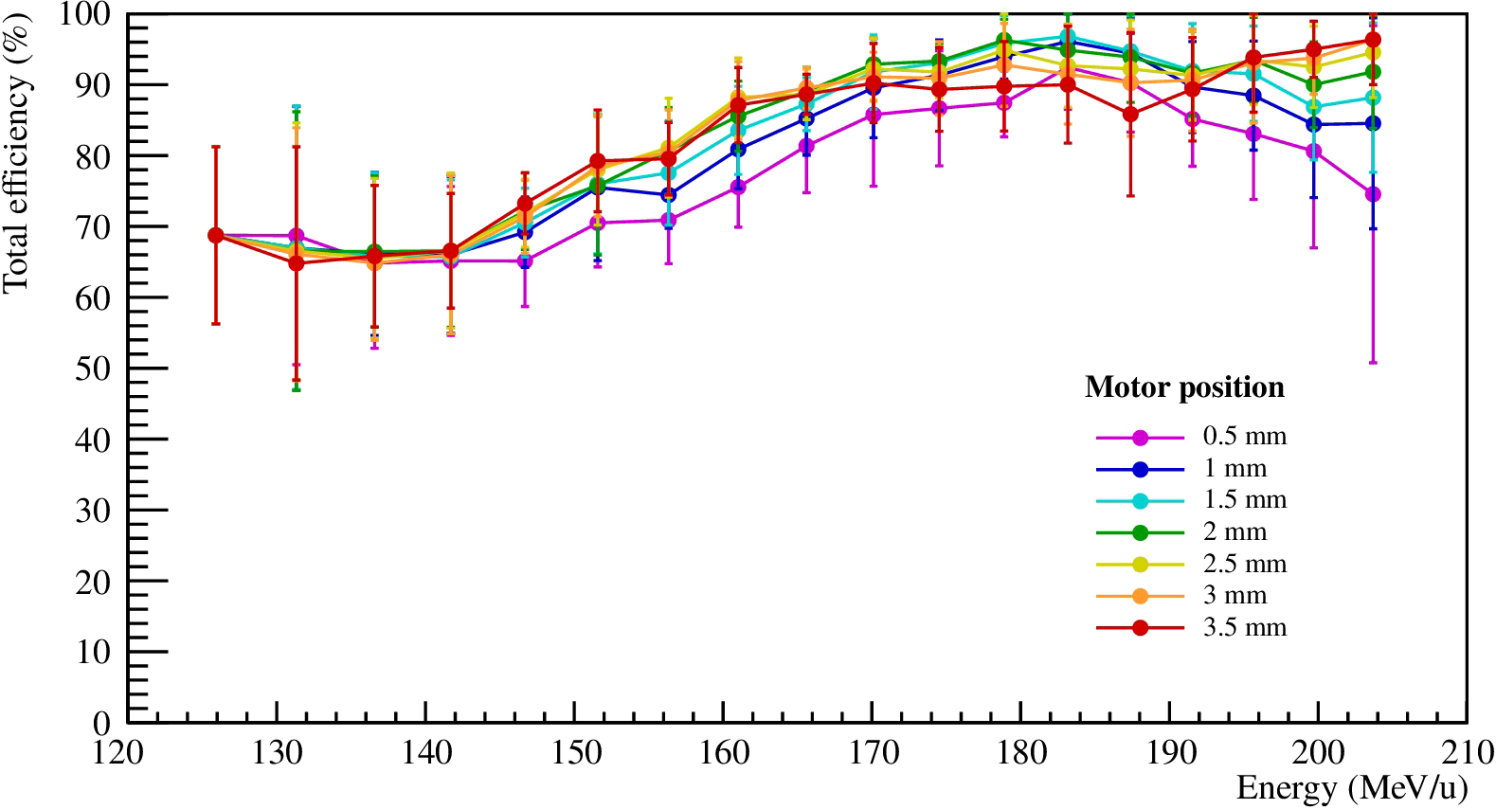
Total efficiency as a function of the primary beam energy for *thr_diff_* = 2·RMS and different motor positions *pos_m_*. The results were computed for the five repeated measurements and the error bars represent the RMS.

As explained in Section 2.2.4, the difference in were computed and compared to a threshold *thr_diff_* of 2·RMS to categorize the beam spots as delivered or not delivered as planned, depending on the set position of the motor *pos_m_*. In this section, a certain amount of beam spots were selected at the edge of the PMMA sphere target, as depicted in **Figure 3**. The goodness of the method was assessed by computing the total efficiency, defined as the ratio of the correctly categorized beam spots and the total number of beam spots in this category. **Figure 10** shows the total efficiency as a function of the primary beam energy and was computed from the iso-energy layers extracted from the 3D treatment plan. The results were computed from the five repeated measurements and the error bars represent the RMS. **Table 2** shows the total efficiency for all iso-energy layers of the 3D treatment plan computed for the five repeated measurements and the error is represented by the RMS.

**Table 2:** Goodness of the method for edge density gradients due to motion.

All iso-energy layers	
Motor position (mm)	Efficiency (%)
0.5	78.4 ± 1.7 1
1.0	82.3 ± 1.3
1.5	83.9 ± 1.4
2.0	84.6 ± 1.8
2.5	84.5 ± 1.7
3.0	84.0 ± 1.3
3.5	83.2 ± 1.6

The total efficiency for *thr_diff_* = 2·RMS and different motor positions *pos_m_* was calculated for all iso-energy layers. The results were computed from the five repeated measurements and the error is represented by the RMS.

The total efficiency for *thr_diff_* of 2·RMS for 131.32 MeV/u varies between 55 and 77%, while for the higher iso-energy layers of 170.11 and 195.65 MeV/u, the total efficiency stays above 85%. As previously explained, the differences between the static_in and moving case for low energy beams are small compared to higher energy ones. The ion beams that stop in the first half of the PMMA target from the planned delivery suffer generally smaller density gradients and smaller range shifts even during motion. Instead, for higher energy beams, larger range shifts are more likely to occur due to high density gradients. Therefore, the present method has a better efficiency for higher energies. The total efficiency for the selection including all energies is individually shown in **Figure 10** and is on average around 80% for all motor positions. As expected, the method has a higher efficiency for a volume comprising the ion beams that produce larger range shifts due to motion and have a higher likelihood to produce fragments with enough energy to leave the phantom and reach a tracker.

### 3.5 Detection of motion phases

**Figure 11:**
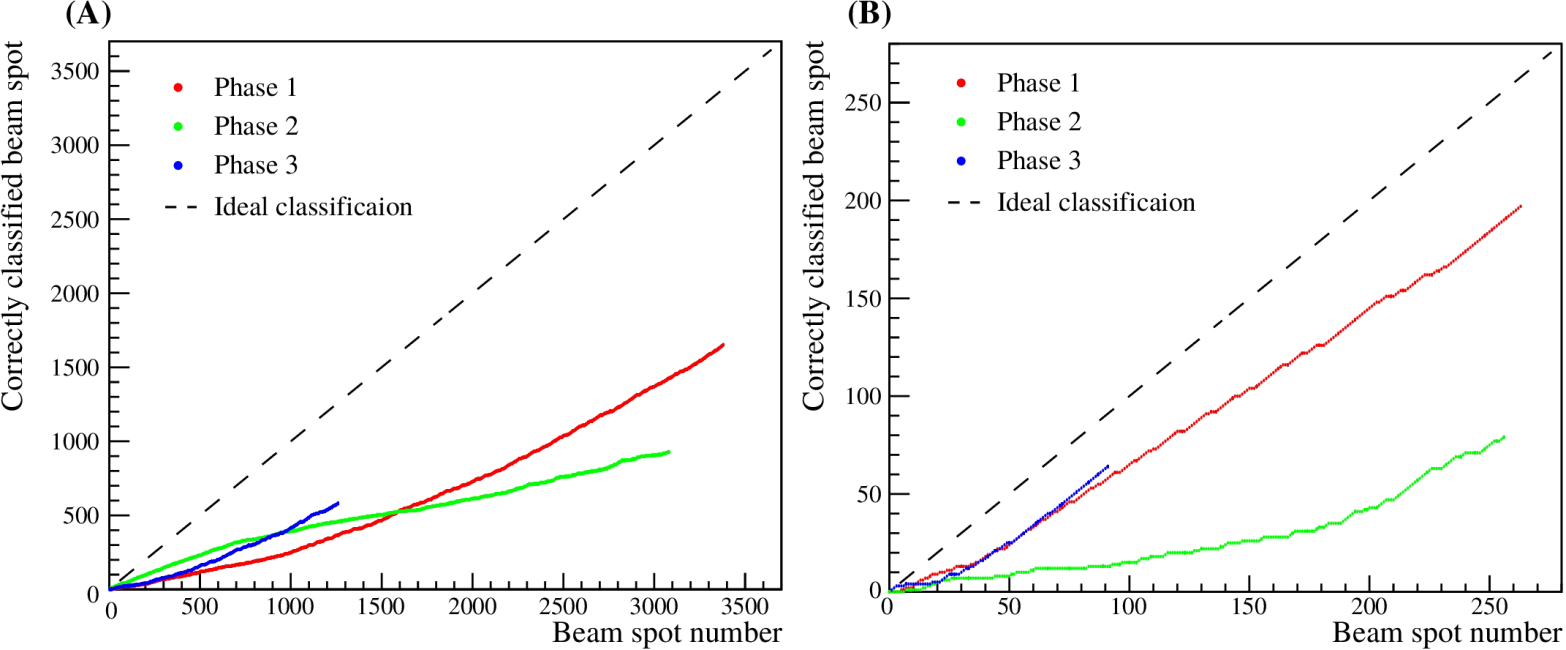
Relation between the correctly classified beam spots as a function of the beam spot number. Panel **(A)** shows the results for the selected beam spots of the 3D treatment plan, while panel **(B)** shows the results for all the beam spots.

The relation between the correctly classified beam spots as a function of the beam spot number was computed for Phase 1, Phase 2 and Phase 3, and is shown in **Figure 11**.

The four cases (undefined, Phase 1, Phase 2 and Phase 3), determined by the measurements, were related to the true motion phases via the motor logfile. In the present study, the planned overshoot – beam spots that do not hit the PMMA sphere due to the treatment planning – were excluded. For the iso-energy layer plans of 131.32, 170.11 and 195.65 MeV/u, a total of 4.2%, 12.7% and 0% were planned overshoot, respectively, and 8.2% for the 3D treatment plan. For all delivered treatment plans, Phase 1, Phase 2 and Phase 3, comprised around 44%, 38% and 18% of the considered total beam spots, respectively. In **Table 3**, the percentage of correctly labeled beam spots from the measurements, compared to the total number of beam spots of Phase 1, Phase 2 or Phase 3, given by the motor logfile, are shown for the three iso-energy layer plans. In **Table 4**, the results are shown for all the beam spots of the 3D treatment plan as well as for the selected beam spots as described in Section 2.2.4.

**Table 3:** Percentage of correctly associated beam spots from the CMOS sensor measurements compared to the total number of beam spots selected via the motor logfile for the three iso-energy layer plans (131.32, 170.11 and 195.65 MeV/u).

131.32 MeV/u				
vertex motor	undefined (%)	Phase 1 (%)	Phase 2 (%)	Phase 3 (%)
Phase 1	65.7 ± 2.9	30.1 ± 3.7	1.4 ± 0.8	2.7 ± 1.6
Phase 2	60.5 ± 1.8	21.1 ± 2.3	2.5 ± 0.9	15.9 ± 2.2
Phase 3	58.3 ± 4.1	0.5 ± 1.0	2.6 ± 1.1	38.3 ± 4.5

170.11 MeV/u				
vertex motor	undefined (%)	Phase 1 (%)	Phase 2 (%)	Phase 3 (%)
Phase 1	19.2 ± 2.0	67.3 ± 2.5	12.0 ± 1.7	1.6 ± 0.8
Phase 2	19.4 ± 2.6	26.0 ± 3.7	32.4 ± 3.7	22.2 ± 1.9
Phase 3	19.0 ± 2.3	2.1 ± 1.8	63.9 ± 4.2	14.2 ± 4.5

195.65 MeV/u				
vertex motor	undefined (%)	Phase 1 (%)	Phase 2 (%)	Phase 3 (%)
Phase 1	16.9 ± 9.2	75.6 ± 10.2	7.5 ± 1.5	0 ± 0
Phase 2	11.7 ± 6.4	36.4 ± 6.7	46.1 ± 4.5	5.7 ± 1.1
Phase 3	11.7 ± 7.3	4.2 ± 1.6	48.8 ± 10.4	35.3 ± 6.5

**Table 4:** Percentage of correctly associated beam spots from the CMOS sensor measurements compared to the total number of beam spots selected via the motor logfile for the 3D treatment plan.

All beam spots				
vertex motor	undefined (%)	Phase 1 (%)	Phase 2 (%)	Phase 3 (%)
Phase 1	24.6 ± 0.3	48.7 ± 0.8	19.9 ± 0.6	6.8 ± 0.3
Phase 2	23.9 ± 1.0	25.5 ± 0.7	28.9 ± 0.8	21.6 ± 1.1
Phase 3	25.5 ± 1.2	7.4 ± 0.6	20.5 ± 1.2	46.6 ± 1.1

Selected beam spots at the edge of the PMMA sphere target				
vertex motor	undefined (%)	Phase 1 (%)	Phase 2 (%)	Phase 3 (%)
Phase 1	2.9 ± 1.1	74.2 ± 3.2	18.5 ± 2.9	4.4 ± 1.3
Phase 2	2.8 ± 0.5	10.7 ± 1.2	33.5 ± 4.0	53.0 ± 3.9
Phase 3	2.9 ± 1.5	3.6 ± 1.9	15.7 ± 5.1	77.8 ± 5.2

The results are shown for all beam spots and for the beam spots selected at the edge of the PMMA sphere target described in Section 2.2.4.

The relation between the computed vertex differences and the motion phases for the three iso-energy layer plans show that 25% of the beam spots are categorized as undefined for the all the beam spots for 3D treatment plan, and 3% for the selected beam spots. The undefined measurements are more likely for low energy beams, however, a certain amount of beam spots are undefined because of small range shifts even during motion. The correct association of Phase 1, Phase 2, and Phase 3 of the CMOS measurements compared to the motor logfile is 48.7 ± 0.8%, 28.9 ± 0.8% and 46.6 ± 1.1%, respectively, for all beam spots of the 3D treatment plan. The highest agreement between measured phases and motor logfile is found for the preselected beam spots at the edge of PMMA sphere with 74.2 ± 3.2, 33.5 ± 4.0 and 77.8 ± 5.2 for Phase 1, Phase 2, and Phase 3, respectively. This behavior is well seen in **Figure 11**, where the correctly classified beam spots show a stronger relation for the selected beam spots at the edge of the PMMA sphere than for all the beam spots of the full 3D treatment plan.

## 4 Discussion

In the present study, a clinic-like treatment plan of 2 Gy, based on a synthetic CT of the used phantom, was generated and a statistical analysis of the reconstructed vertices was performed. As explained in Section 3.1, the number of reconstructed vertices is small compared to the number of delivered primary carbon ions. However, differences of vertex distributions due to high density gradients are evaluated and, therefore, high statistics and positional accuracy are not a necessity.

The presented results from the vertex distributions and positions in *z* were used to assess the feasibility of tracking target motion. Two reconstruction methods were investigated, where the vertices were computed with and without taking into account multiple Coulomb scattering. The differences between the static_in and static_out cases showed that the less realistic and simpler reconstruction method was better suited for this study, yielding stronger differences due to less blurring of the resulting distributions.

The detection of high density gradients was performed after reconstructing the computed vertices in *z* of the moving case, computing the difference with the reference static_in case, and comparing the difference to a threshold value for a selected volume, chosen to consider beam spots that produce larger range shifts during motion (Section 3.4). A method efficiency of 83.0 ± 1.5% and 92.0 ± 1.5% was assessed for the selected scan spots and for half of them containing only higher energy beams with a Bragg peak position behind the center of the PMMA sphere, respectively. For this, the differences of the computed vertices in *z* between the moving and static_in cases and between the moving and static_out cases were calculated and used to relate the reconstructed vertices to the target motion. The resulting relations between the computed vertex differences showed that 25% of the scanned beam spots could not be classified when considering all beam spots, and 3% when considering selected beam spots at the edge of the PMMA sphere target. The correct association of Phase 1, Phase 2, and Phase 3 of the CMOS measurements compared to the total number of beam spots related to the motor logfile is 48.7 ± 0.8%, 28.9 ± 0.8% and 46.6 ± 1.1%, respectively, for the 3D treatment plan. However, for a selected subset of beam spots that produce large range shifts during motion, the method could correctly associate the different phases with 74.2 ± 3.2%, 33.5 ± 4.0% and 77.8 ± 5.2% for the three different phases, respectively. The presented results, especially for the case using a preselected set of beam spots inducing large differences in case of motion, show that the proposed method would be suitable to monitor the performance of, e.g., indirect tumor tracking techniques and intervene in case of excessive drift between real and assumed tumor position.

The main limitations, excluding using a more realistic scenario, which should be addressed for future experimental campaigns, were the precision of the motor position during the experiment, the reliance on a synthetic CT and the general alignment of the measurement setup. The motor positions were recorded every few hundred microseconds and showed an increasing drift of up to ∼ 100 µm between planned and actual position with increasing time for the longest measurements. The relation between the vertex position results and the motor position could be improved with a higher resolution of the recorded time and a drift compensation for the motor. Treatment planning was performed based on a synthetic CT using theoretical density values for the phantom materials as well as dimensions as designed in its technical drawings. Therefore, small differences in material density or geometry of the produced phantom are not taken into account. A treatment planning CT of the phantom with added fiducial markers could help to mitigate these deviations. Additionally, the alignment of the experimental setup purely based on the in-room laser system could be improved by performing radiographic positioning to further increase the accuracy of the phantom positioning. Currently, the experimental setup comprised four CMOS trackers (three sensors each), placed behind the target at a certain distance and several angles that were optimized for a pencil beam and a single energy carbon ion irradiation. However, there are several ways to optimize significantly the performance of the experimental setup without changing the detector setup. Distances and angles of the trackers could be optimized for a complete 3D treatment by spreading the perpendicular bisector of each tracker to the expected extent of the region of interest in *z*, and thus increase the effectively monitored volume. However, the amount of produced secondary charged particles is limited by the number of delivered primary carbon ions as calculated by the treatment planning system and, therefore, direct setup optimizations can only increase the ratio of detected fragments. To drastically increase the number of detected secondary charged particles, more trackers and/or larger area sensors covering a bigger solid angle behind the target, need to be employed. In general, Monte Carlo simulations should be used to predict the optimal placement of the trackers as well as the vertex positions in case the treatment is delivered as planned and for the extreme cases where the treatment is not. However, currently all presented data were reconstructed offline, which only would allow the assessment of the goodness of a treatment after it was delivered. In a next step, the presented approach could be used as an interlock in combination with other motion monitoring and mitigation strategies to stop the beam in case of excessive range deviations. However, stopping an irradiation inevitably leads to the loss of information by the presented technique. In the best case scenario, the presented approach could be improved so that the provided information could be used for real time compensation of the treatment.

Regardless of the chosen optimization steps, precise Monte Carlo simulations are necessary to fully optimize future experimental setups and the presented results can be used to benchmark these 3D- or 4D simulations. Afterwards, additional experimental campaigns should be carried out to assess the performance of the optimized method employing more realistic phantoms.

## 5 Conclusion

This work presented a promising, non-invasive technique for 4D monitoring of high density gradients for the case of carbon ion beam therapy for a simplified lung tumor case. CMOS pixel sensors were used to detect secondary charged particles and to reconstruct their trajectories and interaction points. The reconstructed vertex positions were computed and used to verify a treatment delivery on a per scan-spot basis. The reliability of the concept to predict the motion phase showed good results but can be significantly improved by further optimizing the experimental setup. Strong density gradients were detected in 83.0 ± 1.5% of the cases for the full 3D treatment plan, and in 92.0 ± 1.5% for ion beam energies creating large overshoots, which can induce overdosage in healthy tissues and organs at risk surrounding the tumor. Additional investigations need to be carried out by using Monte Carlo simulations for setup optimizations.

## Data Availability

The raw data supporting the conclusions of this article will be made available by the authors, without undue reservation.
